# QTLs and Genes for Salt Stress Tolerance: A Journey from Seed to Seed Continued

**DOI:** 10.3390/plants13081099

**Published:** 2024-04-14

**Authors:** Keshav Tiwari, Sushma Tiwari, Nivesh Kumar, Shikha Sinha, Saraswathipura L. Krishnamurthy, Renu Singh, Sanjay Kalia, Nagendra Kumar Singh, Vandna Rai

**Affiliations:** 1 Pusa Campus, ICAR-National Institute for Plant Biotechnology, New Delhi 110012, India; 2 Divison of Crop Improvement, ICAR—Central Soil Salinity Research Institute, Karnal 132001, India; 3 Department of Biotechnology, Ministry of Science and Technology, New Delhi 110003, India

**Keywords:** salinity stress, ionic homeostasis, tissue tolerance, rice

## Abstract

Rice (*Oryza sativa* L.) is a crucial crop contributing to global food security; however, its production is susceptible to salinity, a significant abiotic stressor that negatively impacts plant germination, vigour, and yield, degrading crop production. Due to the presence of exchangeable sodium ions (Na^+^), the affected plants sustain two-way damage resulting in initial osmotic stress and subsequent ion toxicity in the plants, which alters the cell’s ionic homeostasis and physiological status. To adapt to salt stress, plants sense and transfer osmotic and ionic signals into their respective cells, which results in alterations of their cellular properties. No specific Na^+^ sensor or receptor has been identified in plants for salt stress other than the SOS pathway. Increasing productivity under salt-affected soils necessitates conventional breeding supplemented with biotechnological interventions. However, knowledge of the genetic basis of salinity stress tolerance in the breeding pool is somewhat limited because of the complicated architecture of salinity stress tolerance, which needs to be expanded to create salt-tolerant variants with better adaptability. A comprehensive study that emphasizes the QTLs, genes and governing mechanisms for salt stress tolerance is discussed in the present study for future research in crop improvement.

## 1. Introduction

The rapidly increasing world population and the potential impact of climate change necessitate an increase in agricultural production by 87% of the current output by 2050, especially for food crops like wheat, rice, maize, and soy [1]. Salinity stress, one of the various abiotic stresses, is a significant barrier in many rice-growing countries, particularly in tropical coastal areas with predominately rice-based farming systems. More than 45 million hectares of irrigated land worldwide have been documented to be negatively impacted by salinity stress. It was recently identified that salt affects 424 MHa of topsoil and 833 MHa of subsoils. Among these, 85% of topsoils and 62% of subsoils are saline. Increasing salinity levels in the soil render about 1.5 million hectares agriculturally unfavourable each year [2]. Particularly in South and Southeast Asia’s coastal regions, which produce 65% of the world’s rice, increased floods and salt intrusion into inland freshwater renders many areas abandoned to fallow, which drastically reduces the rice production in these areas [3]. Furthermore, because current rice cultivars are naturally sensitive to salinity stress, particularly during the seedling and reproductive stages, tolerant varieties should have both higher yield potential as well as stability. 

Sustainable crop production in salinity-affected soils can be attained by practising two key tactics, including a biological approach focussing on exploiting and/or developing varieties capable of tolerating excessive salt levels and a technological strategy for reclamation, drainage, and irrigation with high-quality water. It is thus crucial to know the characteristics of salt-affected soils. Saline and sodic soils are two types of salt-affected soils. Salinity is a measurement of the number of soluble salts in the soil that inhibit proper crop growth. Significant amounts of sodium, calcium, magnesium chlorides, and sulphates are present in saline soils. Salinity is frequently inferred indirectly from a soil extract’s electrical conductivity (EC). The electrical conductivity of saline soils is greater than 4 dS/m. Na^+^ concentrations of above 15% at the exchange sites of the negatively charged clay particles are a defining characteristic of sodic soils. The exchangeable Na^+^ percentage (ESP) of a saturated soil extract rises above 8.5 at such high levels. These soils have low hydraulic conductivity and excessive amounts of carbonates and bicarbonates of Na^+^. While these two categories account for a significant fraction of salt-affected soils globally, there are some transition formations as well; saline-sodic soils exhibit characteristics of both saline and sodic soils. The term salt affected is a broad term for lands which are saline and/or sodic. With a limitation of arable land, the challenge to feed the burgeoning population calls for the amalgamation of different approaches. In order to develop new tolerable cultivars with higher yield potential and stability under various growing conditions, it is necessary to have a deeper understanding of the process underlying the high salt stress. To find salinity tolerance genes for the genetic improvement of rice varieties, we emphasise in this review an integrated approach combining physiological, biochemical, and molecular studies.

## 2. Possible Effect and Mechanism of Salinity in Rice

Osmotic stress and ionic stress are the two main problems that plants must deal with when exposed to excessive salinity stress. When the salt content of the water outside the root increases, osmotic stress occurs immediately. The increased salt level outside the root causes inhibition of water uptake, lateral bud development, and cell expansion [2]. Later, the ionic phase develops when the level of toxic ions such as Na^+^ accumulates in the plants above a threshold level, especially in the leaf, leading to increased leaf mortality with necrosis and chlorosis as well as a reduction in the efficiency of crucial biological metabolism, such as photosynthesis [4,5]. Recent genetic, molecular, and physiological studies have increased the knowledge and information about how plants overcome and cope with detrimental effects caused by salinity stress. Plants respond with various molecular approaches in response to salinity [6]. Below are some of the responses primarily followed by rice plants.

## 3. Stress Sensing and Signal Sensing

**Figure 1:**
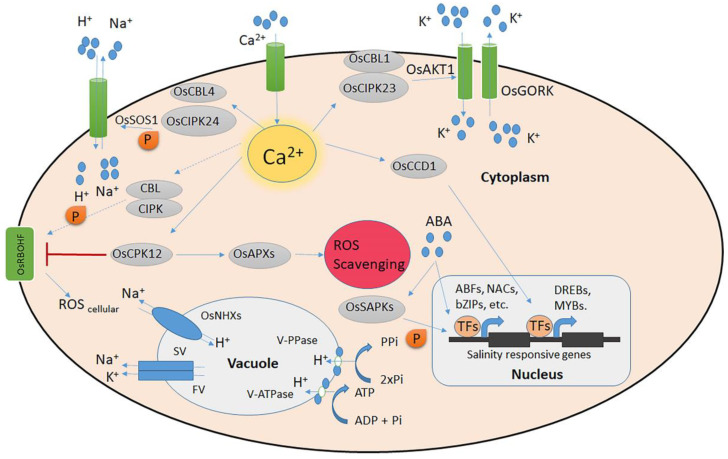
Rice signalling systems for salt stress. The Na^+^/K^+^ ion homeostasis and the detection of salt-induced Ca^2+^ signals are controlled by the CBL–CIPK calcium signalling network. The CBL/CIPK protein kinase complexes are activated by salt, which modifies the function of the Na^+^/H^+^ antiporter *OsSOS1* and the K^+^ transporter *OsAKT1*. Under conditions of high salt stress, *OsCCD1* can bind cellular Ca^2+^ and increase transcription factor levels. The ATP-controlled *OsGORK* is a K^+^ efflux channel under salt stress. At the plasma membrane, *OsRBOHA/F* is involved in the formation of ROS, while *OsAPXs*, controlled by *OsCPK12*, scavenge accumulated ROS. *OsNHXs* powered by either V-ATPase or V-PPase slow-vacuolar (SV) and fast-vacuolar (FV) ion channels and H^+^ pumps are all involved in regulating ion homeostasis in the vacuole under high salinity. Solid arrows denote established direct regulation. Uncertain paths that need to be further investigated are indicated by dashed lines.

To adapt to salt stress, plants detect and translate osmotic and ion signals into the interiors of their cells, which are followed by a modification of their cellular properties. As of yet, no specific Na^+^ sensor or receptor has been identified in plants [7]. However, extensive study has been conducted on the salt overload sensitive (SOS) signalling system and the calcineurin B-like (CBL)/CBL-interacting kinase (CIPK) pathway in Arabidopsis. A salt-induced rise in cytosolic Ca^2+^ activates the SOS2-SOS3 protein kinase complex by phosphorylating and enhancing the activity of SOS1, a plasma membrane Na^+^/H^+^ antiporter [8]. The function and connections between the genes *OsSOS1*, *OsSOS2*, *OsCIPK24*, and *OsSOS3/OsCBL4* have been studied in rice. Together, *OsCBL4* and *OsCIPK24* activate *OsSOS1* [9]. It has been proposed that the CBL10–CIPK24 complex in Arabidopsis constitutes a unique salt-tolerance pathway that regulates vacuolar Na^+^ sequestration [10]. The *OsCBL1–OsCIPK23* complex regulates K^+^ absorption by *OsAKT1* in roots [11]. Additionally, it was shown that the bulk of the rice CBL and CIPK genes respond transcriptionally to abiotic stress, such as salt [12]. These findings imply that more investigation into CBL–CIPK signalling networks in response to salt stress is necessary (Figure 1).

In addition to CBLs and CIPKs, calcium-dependent protein kinases (CDPKs) also control the downstream component of calcium signalling pathways. A total of 29 CDPK genes have been identified in the rice genome, some of which are associated with the response to salt stress. Rice’s ability to tolerate cold and salt/drought is positively regulated by *OsCDPK7* [13]. *OsLEA3*, *OsNAC6*, *OsNHX1*, and *OsSOS1* were among the genes that were induced by ABA and salt when *OsCPK21* was overexpressed [14]. Plants overexpressing *OsCPK12* (*OsCPK12-OX*) showed improved salt tolerance and less hydrogen peroxide (H_2_O_2_) build-up in the leaves. *OsCPK12* positively regulated ROS detoxification via promoting the expression of *OsAPX2* and *OsAPX8*, according to a gene expression study [15]. Many calmodulins (CaM) and CaM-like (CML) proteins, such as *OsCam1- 1*, *OsCML4*, *5*, *8*, and *11*, and *OsMSR2,* were found to be associated with salt tolerance [16,17,18]. A novel, small calcium-binding protein 1 (*OsCCD1*) that is activated by osmotic stress, salt stress, and a calcium-mediated ABA signal can increase rice seedlings’ tolerance to osmotic and salt challenges [19].

Salinity of the soil also causes osmotic stress on roots. An osmotic stress sensor in Arabidopsis was identified as the hyperosmolality-gated calcium-permeable channel *OSCA1* encoded by reduced hyperosmolality-induced [Ca2^+^]_i_ increase1 [20]. A homolog of *AtOSCA1* was reported in rice (*OsOSCA1.2*) [21]. The cryo-electron microscopy structure led to the identification of the function and structure of *OsOSACA1.2,* which resulted in the development of a model in rice for mechanosensitive mechanisms of salt stress tolerance. Mechanosensitive sensors, such as proteins from the two-pore potassium channel (TPK) family and mechanosensitive channel-like (MSL) families, could detect the drop in cell turgor pressure caused by salt stress [22].

## 4. ROS Scavenging and Antioxidant Signalling

Numerous stress-related genes are produced as a result of sensing and signal transduction in the cytosol [23]. Many important proteins, including those involved in root growth and ROS scavenging, are produced by these genes [24]. Several biochemical and chemical processes, such as Haber–Weiss–Fenton reactions [25], excess energy in the mitochondrial electron transport chain (ETC) [26], upregulation of NADPH oxidase in the plasmalemma [27], and alterations in the cytosolic ascorbate-glutathione cycle [28], can result in the production of ROS in roots.

The primary ROS in plants is hydrogen peroxide (H_2_O_2_), superoxide anion (O^−^_2_), singlet oxygen (^1^O_2_), and hydroxyl radicals (OH). These compounds are also formed in peroxisomes, chloroplasts, mitochondria, and by a number of apoplastic sources. Low rates of photosynthesis caused by high salinity also increase the formation of ROS in chloroplasts [29]. In reaction to environmental stimuli, ROS serve as significant signalling molecules. The plant NADPH oxidases, known as respiratory burst oxidase homologs (RBOHs), are essential signalling nodes in ROS signalling pathways. Due to the fact that plant RBOHs include two Ca^2+^-binding EF-hand motifs and phosphorylation target sites in their N-terminal extension, it is possible to combine calcium signalling with ROS production in these cells [30]. By activating a variety of ROS-sensitive ion channels and disrupting the balance of ions in the cells, the accumulation of ROS brought on by stress destroys important cellular structures. To combat stress, plants have evolved enzymatic and non-enzymatic ROS-scavenging mechanisms [31]. The Halliwell–Asada system, commonly known as the ascorbate–glutathione (AsA–GSH) recycling pathway, is at the centre of redox homeostasis and plays a significant part in H_2_O_2_ scavenging in plants [32].

Expression of the genes encoding ascorbate peroxidases (APX), catalases (CAT), type III peroxidases (POD), and glutathione peroxidases (GPX) may be up- or downregulated as a result of salt and osmotic stress [24,33]. By catalysing the conversion of H_2_O_2_ to H_2_O and O_2_, the heme-peroxidase (class I) enzyme ascorbate peroxidase (APX) plays a significant role in scavenging ROS [34]. Eight APX isoforms exist in rice, with two being found in the mitochondria, two in the cytosol, two in the chloroplast, and two in the peroxisomes [35]. A high number of cytosolic APX isoforms are present in plants, and these enzymes play a significant role in the leaves’ defence against abiotic stress. In response to stress, *OsAPX2* may regulate the concentration of H_2_O_2_ in the cytosol, and overexpressing *OsAPX2* enhanced rice’s ability to withstand salt. In rice roots, NaCl induces *OsAPX8* expression that is more closely linked to Na^+^ than Cl^−^ or osmotic factors and is mediated by a build-up of ABA rather than H_2_O_2_ [36].

Another antioxidant offering stress relief is reduced glutathione (GSH). Exogenous GSH boosts endogenous GSH levels and activates glutathione reductase (GR), APX, and superoxide dismutase (SOD) to promote salt tolerance [37]. Glutathione reductase (GR), which catalyses the simultaneous oxidation of nicotinamide adenine dinucleotide phosphate and the reduction of oxidised glutathione (GSSG) to GSH, is an essential component of the AsA–GSH cycle (NADPH) [38]. Rice has RGRC2, which is significantly stimulated by abiotic stressors connected to ABA, such as salinity [39]. One cytosolic GR (*OsGR2*) and two chloroplastic GRs (*OsGR1* and *OsGR3*) have been discovered in rice [33,40]. *OsGR3* conferred salt resistance via controlling GSH redox status in the chloroplasts and mitochondria, respectively [41].

## 5. Variation in Salt Tolerance between Species 

Salinity stress tolerance varies over a wide range from extremely sensitive glycophytes to highly tolerant halophytes for different plant species. Cereal crops are glycophytes with differing degrees of tolerance and mechanisms to tolerate salinity stress. For instance, wheat (*Triticum aestivum*), one of the three most significant cereal crops in the world, has a moderate resistance to saline stress. Maize (*Zea mays*) is less tolerant compared to wheat, while rice is susceptible to salinity stress conditions [2]. As soil salinity levels reach 15 dS m^−1^ (about 150 mM NaCl), rice cultivars die before maturity while wheat cultivars selected for yield under water-limiting conditions produce a reduced yield of not less than 50%.

Plants are adversely affected by salts present in the soil both outside the roots as well as by salts that are taken up by plants. Subsequent growth reduction can be measured immediately or up to several days to weeks. The primary effect of salinity stress is stomatal closure resulting in increased leaf temperatures and inhibited shoot elongation [2]. This has been categorised as the ‘osmotic phase’. These responses were not just due to the salts affecting water potential [42] and are probably best described as a ‘shoot-salt-accumulation-independent effect’. Munns and Tester reported that a prolonged salinity phase subsequently leads to very high Na^+^ and Cl^−^ concentrations, resulting in premature senescence in older leaves termed the ‘ionic phase’ [2]. The gradual accumulation of salts in this phase up to toxic levels and intolerance of shoots to these accumulations inhibits plant growth and causes leaf senescence. In order to prevent hazardous concentrations of Na^+^ and Cl^−^ in the cytoplasm, compartmentalization of these ions is necessary for plants to grow new leaves at a pace greater than senescence. In one study, an experiment was conducted with two rice genotypes that were significantly different in Na^+^ uptake rates and degrees of salt tolerance. In the ‘Phase I’ response after soil salinization, both genotypes exhibited appreciable growth reduction in the first 3–4 weeks due to the initial osmotic stress. After 4 weeks, the genotypes exhibited different responses in ‘Phase II’; the genotype with a lower Na^+^ uptake rate survived until maturity despite showing a reduction in growth and a reduced growth rate compared to the controls under non-saline conditions, while the genotype with a higher Na^+^ uptake rate underwent a significant reduction in biomass and, consequently, many plants died. This phase response was characterised by the differences in the inherent abilities of the genotypes to cope with increased Na^+^ and Cl^−^ concentrations [43]. 

## 6. Genetic Resource–Land Races, Improved Varieties, and Wild Relatives

Selection for higher yield potential during domestication of rice from wild species to cultivated varieties led to significant loss in genetic diversity from the rice gene pool. In comparison to wild rice, Sun et al. found that the number of alleles in farmed rice was reduced by 50–60%. This calls for broadening the rice gene pool through breeding initiatives using a variety of sources, especially wild rice [44]. A total of 22 wild species and 2 cultivated species, *O. sativa* and *O. glaberrima*, make up the genus *Oryza*, which represents evolutionary diversification of about 15–25 million years [45]. The cultivated species have 2n = 24 chromosomes and AA genome. The wild species have been classified as 17 species so far and have either 2n = 24 or 2n = 48 chromosomes and one of eleven genomes (AA, BB, CC, BBCC, CCDD, EE, FF, GG, KKLL, HHJJ, or HHKK) [46,47,48]. The origin and domestication of Asian cultivated rice (*O. sativa*), which has been the subject of much discussion, is thought to have arisen from the common wild rice (*O. rufipogon* Griff) [49,50]. The perennial wild grass (*O. rufipogon*) is being used as an important resource to genetically improve cultivated varieties, as it has various advantages regarding genetic diversity, superior agronomic traits, and resistance to various biotic and abiotic stresses [51]. Effective transfer of agronomically desired genes from *O. rufipogon* into cultivated rice is the most challenging task due to the diversified genetic basis of cultivated rice as well as the ecological risks caused by transgenic escape. *O. coarctata*, a type of Asian wild rice that is primarily found on the salty coasts of India, can endure prolonged immersion in salt water (20–40 ds m^−1^). Under increased soil salinity, *O. coarctata* maintains tissue homeostasis with the help of individual unicellular hairs also called trichomes. As the concentration of these salts reaches dangerous levels in the tissues, trichomes, which are found on the adaxial surface of leaves, aid in the excretion of the major ions of sodium, chloride, potassium, magnesium, and calcium. Landraces differ significantly in salinity tolerance due to introgression from wild relatives, making them a valuable genetic resource for salinity-tolerant varietal development. Pokkali cultivars have long been recognised as highly tolerant donors and have been used extensively in various genetic and physiological studies. Pokkali refers to a rice cultivation system under saline conditions in Kerala. 

## 7. Genetics of Salt Tolerance and QTL Mapping

Many agronomically important traits in crop plants are polygenic. Each of the contributing genes controls a relatively minor effect and are called quantitative trait loci (QTLs) [52]. Finding these QTLs is crucial for plant breeding. Salinity stress tolerance in plants is polygenically controlled [53], and over the past 20 years, various genes that confer tolerance in plants have been postulated. Numerous studies exemplify that these isolated genes are involved in various processes, for example, in signal transduction pathway and transcription regulation [54,55], ion transport, and metabolic pathways [56,57]. According to Kumar et al., the finding of salt-responsive genes might be accelerated with the availability of a high-quality rice genome sequence and by determining the function of numerous proteins involved in signal transduction, ion transportation, and osmoregulation triggered by high salinity [58]. Chatopadhyay et al. studied the diversity in the Saltol-QTL region in 30 saline tract accessions and validated the findings in 37 breeding lines that were tolerant to salinity at the seedling stage [59].

**Figure 2:**
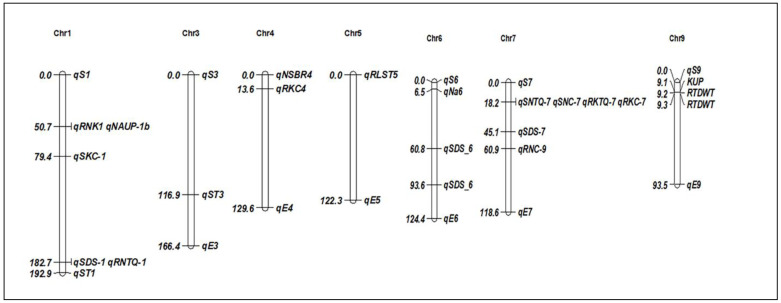
Chromosomal positions of identified QTLs for salt stress tolerance in rice. The QTLs are described in Table 1.

Genetic constituents of salinity tolerance have been characterised in various QTL studies using restriction fragment length polymorphism (RFLP), amplified fragment length polymorphism (AFLP), and microsatellite markers in different breeding populations [60,61]. According to Genc et al., salinity tolerance is not necessarily correlated with a low Na^+^ content in the shoot [62]. Numerous studies have found QTLs linked to rice salinity tolerance (Table 1, Figure 2), particularly in the seedling stage. QTLs have been identified on all 12 chromosomes of rice for salt stress. Out of all the reported QTLs, chromosome 1 has the maximum number of QTLs, and contrastingly, chromosome 11 has the lowest. Several studies have identified Saltol as a major QTL for salt tolerance in the seedling stage. Using a recombinant inbred line (RIL) population derived from Pokkali (salinity tolerant) and IR29, Saltol was located on the short arm of chromosome 1 between RM23 and RM140 (10.7–12.2 Mb) (salinity sensitive). Saltol has been reported to explain 43% of the variance in the shoot Na^+^/K^+^ ratio [60]. Different Pokkali alleles were found in the Saltol region by Thomson et al. They highlighted the potential that the sodium transporter gene SKC1, which is situated at 11.46 Mb and was first reported in Nona Bokra, is the gene responsible for seedling salinity tolerance [61,63]. In an F_2:3_ population made up of the sensitive japonica Koshihikari and the tolerant indica landrace Nona Bokra, Lin et al. discovered a large number of QTLs. Significant QTLs for shoot K^+^ concentration on chromosome 1 (*qSKC-1*), shoot Na^+^ concentration on chromosome 7 (*qSNC-7*), and transport on five chromosomes were among the QTLs they reported [53]. More reports of other QTLs for contributing traits have been found on several chromosomes, including chromosomes 4, 6, and 9 [64,65], and chromosomes 4, 6, 7, and 9 [53]. In a study by Ammar et al., 25 QTLs for Cl^−^, Na^+^/K^+^ ratio, and Na^+^ in leaves at the reproductive stage were found on chromosomes 2, 3, and 8, respectively, in an F_2:3_ mapping population that was produced from the cross between CSR27 (a tolerant indica) and MI48 (a sensitive indica) [66]. Pandit et al. observed eight significant QTL intervals for salt ion concentrations on chromosomes 1, 8, and 12 in the RIL population of the identical cross CSR27/MI48. Additionally, they discovered a QTL on chromosome 8 that was colocalized with one of the important QTL intervals that controlled the SSI for spikelet fertility [67]. Moreover, Cheng et al. discovered twelve QTLs responsible for salt ion concentrations on rice chromosomes 1, 2, 3, 4, 7, and 11 [68]. Due to the laborious and time-consuming phenotyping involved, there have been few investigations on rice’s tolerance to salinity during the reproductive stage [69,70]. Hossain et al. reported several QTLs using an F_2_ mapping population of a cross between Cheriviruppu and Pusa Basmati 1 (PB1) and suggested that salinity tolerance at the reproductive stage was regulated by genomic regions on chromosomes 1, 7, 8, and 10 [71]. Five SSR markers (RM8053, RM345, RM253, RM318, and RM7075) were found by Reddy et al., who evaluated rice accessions against the Dongjin (South Korea) check and distinguished the accessions based on their K^+^/Na^+^ ratios at the seedling stage [72]. QTLs for salt tolerance were also identified in an F_2_ population developed from salt-sensitive Azucena and salt-tolerant Kalarata [73,74]. On chromosome 2 for salt susceptibility index, a unique QTL for grain yield (*qGY2*) was recently discovered that accounts for 45% of the phenotypic variance [75]. In a BC_1_F_2_ population descended from the landraces Wujiaozhan (WJZ) and Nipponbare, a significant QTL (*qGR6.2*) for germination stage salt tolerance was reported [76].

QTL mapping has been heavily utilised in rice breeding programs despite having complex and multigenic characteristics and being labour-intensive, time-consuming, and costly [92,93]. Therefore, the bulked segregant analysis (BSA) method offers a straightforward, quick, and efficient way to locate the genomic regions where markers are related to genes or quantitative trait loci (QTLs) that affect the desired trait(s). In this method, genotyping is only conducted on a pair of pooled DNA samples from two sets of individuals with extreme phenotypes [94,95]. Since 2000, there has been a rapid development of high-throughput genotyping methods based on microarrays and next-generation sequencing (NGS). These techniques coupled with BSA aid identification of numerous genetic markers linked with genes/QTLs of interest. The genetic markers identified are used for mapping genes/QTLs directly. This has led to high-throughput genotyping-assisted BSA becoming increasingly useful to breeders, and studies based on this approach have focussed on qualitative traits [96,97], while studies on quantitative traits (particularly for salinity tolerance in the reproductive stage) are still very limited. Many studies have been reported and used 5–20 extreme bulks, while identification/standardization of the exact number of extreme bulks to pool for identification of QTLs has not been extensively explored. Wolyn et al. were the first to propose the eXtreme Array Mapping (XAM) approach using microarray-based genotyping-assisted BSA and use it for QTL mapping in Arabidopsis [98]. XAM was designed as a time- and cost-effective method for identifying QTLs. Several QTLs underlying resistance to rice blast, grain amylase concentration, and germination rate under low temperatures were mapped by Takagi et al. and Yang et al., respectively, using NGS-assisted BSA [99,100]. Although deep sequencing technologies provide high-resolution genomic and mapping data, the presence of sequencing noise because of variations in reads and irregularities in SNP density still remains a challenge. Various statistical models to remove the effects of noise have been previously used, including the one proposed by Takagi et al., who used the differences in the allelic frequencies. This has been one of the most widely used methods until now [101,102]. Other methods include G-test-based prediction by Magwene, Willis, and Kelly as well as Euclidian distance statistics to measure the divergence [103]. The applicability of these methods was accelerated by the introduction of QTLseqr R packages [104]. A major problem faced during the smoothed statistical analysis is its dependency on population size, effects of QTL, and rate of recombination. Therefore, recently, a statistical tool (R code) has been proposed by De La, Cantó, and Vigouroux [105] to identify the location of QTLs in the bulk of F_2_ lines, even under poor recombination rates. They also developed a simulation approach for the identification of QTLs by building confidence interval statistics. This study may facilitate the selection of NGS-based BSA statistics for crop improvement [106]. 

Rice is susceptible to salt stress at various stages of its growth. Using bulked segregate analysis (BSA) of bi-parental recombinant inbred lines, Tiwari et al. demonstrated a rapid approach for QTL identification during the reproductive stage for salt tolerance (RIL). A 50K SNP chip was used with BSA, which revealed 34 QTL regions in ‘CSR27/MI48’ RIL. These results led to the validation of previously identified QTLs and detected many new ones for further research [77]. 

Insufficiency of studies reporting QTL mapping and map-based cloning during this stage in rice limits our knowledge on salinity stress tolerance. However, some recent progress in this field raises further hope to direct research in this area. For instance, a study by Lei et al. discovered the gene (*OsSAP16*) that encodes a protein of the C_2_H_2_-type zinc finger family that exhibited increased expression in drought-stricken regions. Here, a whole-genome sequencing approach was used to find QTLs in 40 extreme cases of salt-sensitive and 40 extreme cases of salt-tolerant genotypes belonging to an F_2:3_ generation derived through the cross between IR36 (salt-sensitive) and Weiguo (salt-tolerant) plants. The identification of a QTL (*qRSL7*) on chromosome 7 was detected, which was further searched for markers and QTL mapping from 199 individuals belonging to their population. *OsSAP16* was identified as the *qRSL7* candidate gene. Furthermore, the RNA analysis of the parents revealed five differentially expressed genes in the candidate region. These findings point to the potential for *qRSL7* in the improvement of rice varieties against high salt concentrations, particularly those in the bud burst stage. Hence, gene cloning and further validation could aid in developing such varieties through marker-assisted selection [78]. 

A MAGIC (multiparent advanced generation intercross) population was employed for genotyping with a 55k SNP chip, and seven QTLs were identified (Table 1). A QTL for relative root length on chromosome 2 (*qRRL2*) was reported [79]. In addition to QTL discovery, a gene expression study revealed a transcription factor (*LOC_Os01g66280*) as a potential gene for salt stress tolerance. A genome-wide association study (GWAS) was employed for 180 varied accessions of rice and using SSRs, 28 associations with traits were recorded for Na^+^, K^+^, and uptake of Na^+^/K^+^ in the leaves and stem [80]. Therefore, the study unravelled the role of ionic homeostasis as a mechanism for salt tolerance. SNP genotyping was used for 18 advanced breeding lines, wherein a single line had multiple stress tolerance QTLs/genes. Those lines were used for the evaluation of yield stability and efficiency with additive main effect and multiplicative interaction (AMMI) and genotype/genotype–environment (GGE) biplot analysis [107]. Using a reciprocal population developed from salt-tolerant Horkuch and IR29, a major QTL was identified using SNP for leaf total potassium and grain weight [108]. In a study by Goto et al., QTLs for removal of toxic Na^+^ in the leaf sheath were reported at chromosomes 4 and 11 (Table 1), which emphasized the importance of Na^+^ removal from leaf sheaths in decreasing accumulation of Na^+^ in leaf blades [81].

Understanding the molecular basis for genetic variation of agronomically critical complex traits requires QTL detection, followed by narrowing it down further for gene isolation. The most widely used method is to create a group of near-isogenic lines (NILs) for the desired QTL(s). NILs are developed to have a uniform genetic background of the recurring parent while differing only in the genic region having the desired QTL(s). This simplifies the targeted QTL as a Mendelian factor in the NILs. Cloning of the genes underlying the QTLs for rice salinity tolerance is now mostly feasible due to the high-quality rice genome [109,110]. Using a map-based method, the SKC1 gene controlling K^+^/Na^+^ homeostasis in the salt-tolerant indica rice variety Nona Bokra was cloned [64]. The 70 QTLs were mapped for salt tolerance using RIL and DH populations, and two essential salt tolerance genes of rice (SKC1 and DST) have already been cloned. Once the markers for the QTL region are identified, it can be utilised in the breeding program to develop tolerant varieties [111].

## 8. Rice Breeding with Marker Assistance for Salt Tolerance

Marker-assisted selection (MAS) could use various markers for the identification and introgression of QTLs into different rice cultivars. DNA markers, such as simple sequence length polymorphisms (SSLPs), restriction fragment length polymorphisms (RFLP), sequence-tagged sites (STS), and simple sequence repeats (SSRs), have been extensively used in molecular mapping and MAS investigations [112]. For the identification of QTLs linked to salinity tolerance, changes in the allelic distribution within the specified gene of interest provide valuable information on designing and creating novel molecular markers utilized by the MAS technique. Previously, salt tolerance in rice cultivars was improved using markers such as AFLP, SSR, and RFLP to identify QTLs associated with sodium and potassium absorption [113].

In rice, many landraces, such as Pokkali, Nona Bokra, Bundu, Billi Kagga, and Azgo, to name a few, are found to have good salinity tolerance but with a tall height and low yield. Plant breeders are using these landraces to identify and map tolerance-governing traits and transfer the loci to give salinity-tolerant varieties to farmers. Selection and recombination processes release many salt-tolerant varieties (Table 2) involving landraces and traditional varieties, e.g., PSBRc48 (Hagonoy), PSBRc50 (Bicol), NSIC2013Rc336, NSIC2013Rc338, and NSIC2013Rc340 developed from IRRI in the Philippines. Similarly, CSR10, CSR11, CSR13, CSR23, CSR27, CSR30, CSR43, CRdhan405, CRdhan406, Vytilla3, Vytilla4, Panvel2, Usar dhan1, Usar dhan2, and Usar dhan3 were developed in India. Additionally, BRRI Dhan 40, BRRI dhan41, BRRI Dhan 55, and Bina dhan10 were developed in Bangladesh, and OM2717, OM11271, Giza 178, and many more were developed in Vietnam. Egypt has also developed several varieties, such as Sakha104 and Sakha 105. However, the background effect of traditional varieties and the complex nature of the traits involved pose challenges in making significant progress through conventional methods, which has led to an interest in molecular breeding methods. Attempts have been made for more than six decades (from the early 1970s) to enhance the salt tolerance in rice through breeding [114]. Although national and international breeding programs have been running in many countries, the pace has been quite slow, given the few new cultivars released. One of the most prominent was CSR10, which was released from CSSRI, Karnal, India [115]. Salt-tolerant IR64 NILs were created by Ho et al., who demonstrated that the lines exhibited markedly higher tolerance to salinity than their recurrent parent IR64 [116]. Additionally, a broad-spectrum resistance towards various biotic and abiotic factors was observed when introgression of QTLs was performed using gene/QTLs pyramiding [117]. Therefore, it was indicated that gene pyramiding is a potential strategy for reducing salt stress in rice at the moment [118]. A few classical examples are the development of blight-resistant lines in basmati and non-basmati background by incorporating blight genes xa13, Xa21, and xa5; sheath blight resistance QTL, *qSBR11-1*; and the blast-resistance gene Pi54 from elite sources [119,120,121].Similarly, SSR markers for yield-enhancing QTLs have been utilised to increase variety ‘93-11’s yield through MAS, and numerous backcross lines with high yield potential have been created [122]. The use of markers to accelerate varietal development at the seedling stage has led to a preference for MAS. More importantly, because the environment has no bearing on this strategy, there is no need to carry undesirable plants all the way to maturity, allowing for a more efficient breeding strategy. Bulk and pedigree breeding are used for MAS, which is then followed by efficient phenotypic screening.

Many major and minor QTLs have been found and mapped for salt tolerance at the seedling stage on practically all the chromosomes of the rice genome, and these are listed in Section 4. To date, the MAS program has mainly exploited one key QTL, i.e., the large effect Saltol locus for the seedling stage situated on the short arm of chromosome 1, which incorporates seedling stage salinity tolerance. This QTL was identified in a population of recombinant inbred lines (RILs) descended from the IR29/Pokkali, and it exhibited a logarithm of odds (LOD) score of 14.5 while accounting for 81% of phenotypic variation. Saltol governs three salt-related traits: high: K^+^, low Na^+^, and low Na^+^/K^+^ ratio [82]. Markers for the mapped region were developed and effectively used to select positive lines during the selection steps. Bonilla et al. indicated the region flanked by RM23 (~10.7Mb) and RM140 (~12.3Mb) [60], whereas Lin et al. described the QTL for shoot K^+^ concentration flanked by RFLP markers C1211 (~9.81Mb) and S2139 (~11.28Mb) in NonaBokra/Koshihikari population [53]. Numerous salt-tolerant cultivars have been created through MAB, e.g., OM4498 (IR64/OMCS2000) from Vietnam [123].

The use of molecular markers occurs in MAB at three different levels. First, the markers help foreground selection, which is the selection of target alleles whose effects are difficult to gauge phenotypically. Recombinant selection, the second stage, assists in locating plants where recombination close to the target locus results in a target chromosome with a minimal donor segment and a greater tendency toward the receiver segment. The most important step was to reduce the donor section to avoid linkage drag. If the donor is an exotic or wild relative, the drag is greater. The third and final phase is background selection, where plants with recipient-like genomes on all chromosomes but the target allele are chosen using unlinked markers. There may be plants with more or less than the average amount of recipient genome recovery after two backcrosses, which is 87.5% on average. The selection of progenies with maximum recipient background is reached after two or three backcrossing events using molecular markers, reducing the number of generations required to obtain 98 or 99% recurrent parent genome (RPG) [124]. The product developed through MAB is a near-isogenic line (NIL). For example, Swarna-Sub1, a NIL of Swarna with the Sub1 gene for tolerance to submergence at the seedling stage [125] and development of improved Pusa Basmati1 (Pusa 1460), a NIL of Pusa Basmati1 with the Xa13 and Xa21 gene for resistance against blight disease [121]. From the beginning of breeding through varietal release, conventional rice breeding normally takes ten to fifteen years. According to estimates, MAB will save at least three years and provide each country with substantial additional benefits that might vary from $50 to $900 million, depending on the location, abiotic stress, and lag for conventional breeding [126].

Currently, MAB is being utilised to introduce popular, high-yielding varieties from various south and southeast Asian nations. Most of the products/varieties created by IRRI in collaboration with various nations are in the advanced stages of testing and release. A RIL created from the IR29/Pokkali donor line, FL478 (IR66946-3R-178-1-1), was employed as a donor line in most cases. The Saltol region of FL478 has been introgressed from the sensitive parent, IR29, yet the salt tolerance was triggered since IR29’s favourable alleles were present. The size of the Saltol fragment was 10.6 to 11.5 Mb [127]. Microsatellite markers RM8094, RM3412, and RM493 provided the highest results for foreground selection in the majority of backcrossing programs [128]. Linh et al. selected RM 493 and RM3412b for selection in BT7/FL478 [129], whereas Huyen et al. discovered AP3206f and RM3412 to be the most informative foreground markers in transferring Saltol into the Vietnamese variety AS996 [130]. Through the transfer of Saltol, Bangladesh varieties BR11, BRRI Dhan 28, and BRRI Dhan 29 were enhanced for salt tolerance through IRRI and BRRI’s partnership [131]. By transferring Saltol from FL478 to Binadhan-5, Moniruzzaman et al. increased Binadhan-5’s resistance to salinity [132]. IR64-SalTol was developed at IRRI [116]. The Vietnamese variety, BT7, was enhanced by MAB [128]. To clarify the salinity-tolerance metabolic pathways, Mishra et al. employed the salt-tolerant CSR27, salt-sensitive MI48, and their extreme tolerant and sensitive RIL progenies. In their investigation, proteome profiling for tolerant lines confirmed gene colocalization in the salinity tolerance QTL intervals mapped in the RIL population [57]. Rahman et al. screened RILs derived from IR29/Hasawi and identified eight hotspots conferring salinity tolerance across environments colocalised on chromosomes 1, 4, 6, 8, and 12 [83,133].

The impact of biotic and abiotic stressors on the Indian rice variety Naveen was studied by Ramayya et al., who employed marker-assisted backcross breeding (MABC) and marker-assisted forward breeding (MAFB) studies to introduce drought-resistant QTLs like *qDTY1.1*, *qDTY2.2*, and *qDTY4.1* into the Naveen framework. Identification of highly resistant lines possessing drought tolerance was performed without compromising yield under non-stress conditions using extensive selections based on phenotype. These experiments led to the reporting of eight lines from MAFB and twelve lines from MABC with three to six QTLs against various biotic stresses and drought stress (at the reproductive stage) for greater yield in comparison to Naveen. It was found that the combined approach of MAFB and MABC produced better-yielding lines than the application of just MAFB. Thus, multiple resistances, both biotic and abiotic, could be conferred to various rice varieties through combined breeding strategies [134]. 

Salinity tolerance QTLs have been discovered during the reproductive stage and the seedling stage [66,67,135]. In a population of recombinant inbred lines (RILs) resulting from the cross between the salt-tolerant variety CSR 27 and the salt-sensitive variety MI48, Pandit et al. discovered a substantial QTL for spikelet fertility (*qSSISFH8.1*) on chromosome 8. Between marker interval HvSSR08-25 (position 9.27Mb) and RM3395 (position 10.29Mb), MI48 contributed the QTL locations, with a LOD score of 4.17, explaining 8% of the phenotypic variance. The lines produced from a population of BPT5204/CSR27 at IIRR in Hyderabad, India, demonstrate the presence of reproductive stage salinity tolerance. Significant rice cultivars will benefit from the total protection provided by the introduction of both seedling and reproductive stage salinity tolerance QTLs, resulting in increased yields in stressed regions [67].

Another recent study showed SNP-based marker-assisted selection, where the *hst1* gene was introgressed from “Kaijin” germplasm possessing salinity tolerance to “Yukino-mai” (WT), which is a high-yielding variety. A BC_3_F_3_ population (YNU31-2-4) was created by Rana et al. utilising the biotron speed-breeding method. Whole genome sequencing was performed to get high-resolution genotypic data, which showed 93.5% similarity between the BC_3_F_2_ population and the WT. Under normal conditions of growth, they possessed similar agronomic characteristics to the WT; however, upon subjection to salt stress (125 mM NaCl), they unexpectedly exhibited an increased rate of survival along with enhanced biomass in the shoot and root compared with the WT. Other observations in the YNU31-2-4 population under salt stress showed higher assimilation of net CO_2_, a lower decline in yield, improved phenotype in the reproductive stage, and avoidance of accumulation of Na^+^ in shoots at the seedling stage of growth [136]. The salt tolerance mechanism in these populations was physiological and biochemical in terms of higher growth, high water content, and increased proline content under high salinity. The amount of proline provides stress tolerance through the maintenance of cell osmotic balance and protection of cell membranes [137]. It is also involved in reserving organic N_2_ during the stages of stress recovery in plants [138,139,140]. Therefore, the YNU31-2-4 population was suggested to be a promising candidate for the improvement of salt stress under both seedling and reproductive stages of rice cultivars for the maintenance of higher yields even in changing environmental conditions. The Saltol locus was recently transferred to two varieties of temperate japonica, Vialone Nano and Onice, where KASP markers were employed for background and foreground selection. A total of 15,580 SNPs obtained from GBS were used for genetic background recovery [140].

## 9. Meta-Analysis of QTL Associated with Salinity Tolerance

Meta-QTL analysis refers to a process of exploring the complex genetic traits associated with the possible molecular markers to be employed in marker-assisted selection. It offers a higher mapping resolution and a broader allelic coverage [141]. Various complex agronomic traits in plants such as salt tolerance, which are generally affected by genetic and environmental factors, are regulated by QTLs [63]. Therefore, QTL analysis is necessary to comprehend the fundamental genetic variations present in these polygenic traits [142]. Despite identification of many such QTLs, different environment and genetic factors restrict their introgression in rice breeding programs. Nevertheless, cloning of the SKC1 locus in accordance with the *qSKC1* QTL [63] shows the possibility of accurate mapping given that the distance between the two markers is less than 2cM [110]. Additionally, studies on analysis of meta-QTLs have speculated the presence of genes concerning the salt tolerance in rice on 12 chromosomes. Hence, attempts to find the QTLs specifically related to salinity tolerance across varying genetic and environmental conditions will need to be conducted in the near future to improve the marker-assisted breeding program. 

The salt injury score (SES) has been the best indicator for estimating the salt tolerance of genotypes and various other associated morphological traits in plants [143]. As per this score, FL478 has been the best genotype, and IR29 has been the poorest genotype regarding salt tolerance. These genotypes are often used as controls in screening of genotypes grown hydroponically for seedling-stage salt stress resistance in rice. Many studies have recently been performed to validate the genomic loci concerning salinity tolerance. For instance, a study conducted by Prakash et al. identified a microsatellite marker “RM5635” associated with MSQTL4.2, which is around 295.43 kb in size. This marker showed contrasting characteristics to the genotypes associated in the seedling stage of salt tolerance. However, none of the markers could be identified for genotypes concerning salinity stress in the reproductive stage. They utilized 45 QTL-mapping studies and 915 unique QTLs to conduct their trials. Out of these, 49 and 65 QTLs were linked to reproductive and seedling stage salt tolerance in rice. From this study, they identified eight extreme genotypes (highly susceptible and highly tolerant) growing hydroponically for salinity stress (EC~ 10.0 dSm^−1^) in the seedling stage and identified another eight genotypes growing at the reproductive stage, i.e., saline microplot circumstances (EC~ 8.0 dSm^−1^). These genotypes were chosen based on SES scores and SSR markers to validate the hypothesized meta-QTLSs [144]. Later, upon performing gene expression studies on the identified QTLs, downregulation of a gene *(Os04g0423100)* responsible for a protein that acts as a co-factor in various important metabolic processes, including hormonal metabolism, pathogenic responses, stress signalling, etc., [145] as well as a role in auxin and glucosinolate metabolism under stress was observed [146]. This protein is a “monooxygenase” with a FAD-binding domain whose downregulation switches off salt stress signals, which enables the plants to simultaneously avoid the stress response and actively focus on other metabolic pathways [147]. The chlorophyll content of the leaf and sodium and potassium concentrations in the root and shoot are the traits linked to this QTL. Therefore, the discovery and validation of such genomic areas linked to rice plant seedling salinity tolerance open the door to marker-assisted backcross breeding techniques [144]. Additionally, the absence of a strong QTL and adequate QTL-mapping studies for salinity tolerance in rice at the reproductive stage raises the possibility of expanding the investigations for future discovery and validation [143].

Another set of experiments performed by Islam, Ontoy, and Subudhi revealed various candidate genes possessing meta-QTL regions with salt-tolerance characteristics. They performed phenotyping of 56 different genotypes, from which 6 were identified to be associated with salinity stress. Eleven meta-QTLs were identified on chromosome numbers 1 and 2 within this genomic region. This selection was based on the presence of three important features, including SIS, SNC, and SNK [147]. It was already reported that traits such as SNC and SKC are regulated by the same chromosomal region using SSR markers [148]. Based on SIS scoring, examination of rice genotypes at the seedling stage for salinity tolerance identified four meta-QTLs. In times of salt stress, plants’ uptake of Na^+^ and K^+^ ions aids in SIS scoring. Upon response to salt stress, mechanisms such as ion homeostasis, transcription regulation, scavenging of ROS, and stress signalling is initiated in the salt-tolerant rice genotypes. Candidate genes were discovered, including those involved in potassium transporter, pectinesterase, peroxidase, transcription control, and cell wall organization [84]. 

## 10. Conclusions

Plants are negatively affected by the presence of toxic salts in the soil, which leads to subsequent growth retardation. The primary effect of salinity stress is stomatal closure, which results in increased leaf temperatures and inhibits shoot elongation. This is the ‘osmotic phase’. These responses are not just due to the salts affecting water potential but also due to the ‘shoot-salt-accumulation-independent effect’. The roots are the first to come in contact with salt, which triggers myriad reactions, including sensing and signalling, that lead to the induction of a number of genes, such as *OsSOS1*, *OsSOS2*, *OsCIPK24*, and *OsSOS3/OsCBL4*. A gene complex *OsCBL1-OsCIPK23* regulates the absorption and transport of ions, and *OsAKT1* helps in the absorption of K^+^ to protect the cells from the toxic effect of Na^+^. The majority of CBL and CIPK gene expression was regulated in rice. Therefore, more investigation is required to understand the sensing and signalling pathways in rice under salt stress conditions. Due to the complex phenotypic, physiological, and polygenic nature, salinity stress tolerance is significantly affected by ecogeographic locations rendering identification of genes/QTLs/genetic markers difficult for marker-assisted selection (MAS). Using QTL mapping, breeders can find genic areas responsible for differences in the desired trait. The information presented here can be exploited for designing crosses in breeding programs to develop improved lines for salt stress tolerance. The present study showed how DNA markers have been utilised in marker-assisted backcrossing to improve rice varieties so they can withstand high salinity stress. DNA markers, e.g., SSRs and SNPs, can be effectively employed to increase the efficiency and precise introgression of the locus connected with the desired trait. Once the QTL governing the attribute is mapped, the tightly linked markers are identified for the gene/QTL of interest. It is validated in varieties/landraces and utilized accessions and in the population developed for the particular trait. The lost variation during the domestication process from wild rice species to cultivated varieties reduces the rice gene pool. The number of alleles in cultivated rice was reduced by 50–60% in comparison with wild rice, which calls for expanding the gene pool by breeding using diverse sources, especially wild rice. Therefore, it is also imperative to use wild sources as a rich genetic material for further improvement to sustainable agriculture.

## Figures and Tables

**Table 1:** QTLs reported for salt stress tolerance in rice crops with their chromosomal positions.

Mapping Population	Total Number of Markers	Trait Studied	Name of QTL	Chr.	Remarks	Ref.
Nonabokra/Koshihikri 133 F2	161 RFLP	Root and shoot Na^+^, K^+^ uptake and Na^+^/K^+^ concentration	Identified 11 QTLs: qSDS-1, qSDS-6, qSDS-7, qSNC-7, qSNTQ-7, qSKC-1, qRNC-9, qRNTQ-1, qRKC-4, qRKC-7, qRKTQ-7	1, 4, 6, 7, 9	Two significant QTLs with a very large effect, qSNC-7 for shoot Na^+^ concentration and qSKC-1 for shoot K^+^ concentration, explained 48.5% and 40.1% of phenotypic variance, respectively.	[53]
Pokkali/IR29 (RILs) 78 RILs	23	Na^+^ and K^+^ uptake and Na^+^/K^+^ ratio	Saltol	1	Define position of Saltol QTL; RFLP SSR flanking markers: RM23, RM140	[60]
Pokkali/IR29 140 RILs	100 SSR	Na^+^, K^+^ concentration, Na^+^/K^+^ ratio in root and shoot, SES tolerance score, leaf chlorophyll content	Identified 24 QTLs: qPH2, qPH4, qSNC1, qSNK1, qSNK9, qRKC1, qRKC2, qRKC6, qRNK1, qRNK6, qRNK9, qSES4, qSES9, qCHL2, qCHL3, qCHL4, qSES3, qSES12, qSUR1, qSUR2, qSUR12, qCHL1, qCHL1, qCHL12	1, 2, 3, 4, 6, 9, 12	Saltol contributes to Na^+^/K^+^ homeostasis; SKC1 may be the causal gene underlying saltol QTL. Identified a region on chromosome 2 contained a cluster of Pokkali-derived QTLs, including height, root K^+^ concentration, chlorophyll content and survival.	[61]
Nona Bokra/Koshihikari 192 BC2F2 and 2973 BC3F3 NILs	14 AFLP/SSTs	K^+^/Na^+^ homeostasis	qSKC1	1	Isolated SKC1 gene (7.4 Kb) by map-based cloning; Flanking markers of QTL: K159, K061	[63]
IR59462/Nona Bokra/Pokkali//IR4630-22- 2-5-1-3/IR10167-129-3-4 150 F7 NILs	Four	High Na^+^ uptake, K^+^ uptake and Na^+^/K^+^ discrimination	Identified 16 QTLs governing different ion concentrations: QNa, QK1, QK2, QNaK	1, 9, 6, 4	QTLs for the presence of Na and K in the shoots have been located using AFLPs.	[64]
IR4630/IR15324 118 RILs		Na^+^, K^+^ uptake, Na^+^/K^+^ ratio, dry mass production, concentration of ions	Identified 11 QTLs: Chr1; Na^+^ uptake, K^+^ concentration, Na^+^/K^+^ ratio; Chr4: K^+^ uptake K^+^ concentration, Na^+^/K^+^ ratio; Chr6: dry mass, K^+^ uptake, Na^+^ concentration; Chr9: K^+^ uptake	1, 4, 6, 9	QTL for K^+^ uptake with the largest effect was found on chr 9, explaining 19.6% variation AFLP and RFLP markers	[65]
CSR27/MI48 216 (F2/F3) RILs	SSRs	Seedling salt injury score, Na^+^, K^+^, Cl^−^ concentration, Na^+^/K^+^ ratio in leaf and stem tissue at vegetative and reproductive stages	Reported 25 QTLs: qSIS-1.1, qNaLV-3.1, qNaLV-8.1, qNaLV-8.2, qNaLR-2.1, qNaLR-3.1, qNaLR-8.1, qNaSV-1.1, qNaSV-2.1, qNaSV-8.1, qKLV-3.1, qKLR-8.1, qKSV-1.1, qNa/KLR-3.1, qNa/KLR-8.1, qNa/KSV-1.1, qNa/KSV-2.1, qNa/KSV-2.2, qNa/KSV-2.3, qNa/KSV-3.1, qNa/KSV-8.1, qClLV-3.1, qClLR-2.1, qClSV-1.1, qClSV-2.1	1, 2, 3, 8	QTL interval RM563- RM186 on chromosome 3 was the most important as it influenced nine of the seventeen salt tolerance parameters studied	[66]
CSR27/MI48 216 F7 RILs	1058 SSR (598 RM and 460HvSSR)	Na^+^, K^+^ and Cl^−^ ion concentrations in different tissues and salt stress susceptibility index for spikelet fertility, grain weight and grain yield	Identified 9 QTLs: qKLV1.1, qNaSH1.1, qKSH1.1, qNa/KSH1.1, qNaSH8.1, qClLV8.1a, qClLV8.1b, qSSISFH8.1, qNaSV12.1	1, 8, 12	A significant QTL for SSI for spikelet fertility at high salt stress (qSSISFH8.1) was located on chr 8 in marker interval HvSSR8-25-RM3395	[67]
Zaiyeqing8/Jingxi 17 (DH)		Survival days of seedlings on 0.7% NaCl Yoshida solution	Identified 8 QTLs for survival times of seedlings in 0.7% NaCl	1, 2, 3, 7, 8, 12	Major QTL Std on Chr1; flanking markers RG612, C131	[69]
Sadri/FL478 232 F2	155 SSR	plant height, days to flowering, panicle length, no of panicles, spikelet no, 1000 grain weight, grain yield under salinity stress	Identified 35 QTLs: qDTF4.1, qDTF6.1, qDTF10.1, qPH1.1, qPH3.1, qPH5.1, qPH7.1, qPL1.1, qPL2.1, qPL3.1, qPN4.1, qPN6.1, qPN9.1, qSTW4.1, qSTW7.1,qSTW8.1, qSTW9.1, qFRSP2.1, qFRSP4.1, qFRSP6.1, qFRSP10.1, qSTSP3.1, qSTSP7.1, qTSP4.1, qTSP7.1, qTSP9.1, qGY2.1, qGY4.1, qGY6.1, qGY8.1, qSPFR2.1, qSPFR2.5, qSPFR2.10, qTGW5.1, qTGW6.1, qTGW6.8, qTGW10.1	1, 2, 3, 4, 5, 6, 7, 8, 9, 10	Three major QTL clusters were found on chromosomes 2 (RM423–RM174), 4 (RM551–RM518) and 6 (RM20224– RM528) for multiple traits under salinity stress.	[70]
Cheriviruppu/Pusa basmati 1 218 F2/F3	131 SSR	Plant height, tiller no, panicle length grain yield, biomass, pollen fertility, Na^+^/K^+^ ratio, Na^+^ concentration at reproductive stage	24 QTLs: qPH1.1, qPH4.1, qPH7.1, qTN7.2, qTN7.3, qTN8.1, qPL1.2, qPL7.4, qGY2.1, qGY3.1, qGY, qGY12.1, qBM8.2, qPF1.4, qPF1.5, qPF10.1, qPF10.2, qNa1.6, qNa1.7, qNaKR1.8	1, 7, 8, 10	Tight cluster of QTLs on chromosome 1 at position 31.06 Mb novel loci different from saltol and SKC1 at reproductive stage	[71]
Kalarata/Azucena 400 F2	151 SSR	Shoot fresh weight (SFW), Shoot dry weight (SDW), Root dry weight (RDW), Shoot K^+^ concentration (SKC), Root K^+^ concentration (RKC), Shoot Na^+^ concentration (SNC), Root length (RL), Chlorophyll b (CHLB), Root Na^+^ concentration (RNC), SES	qSFW1.1, qSDW1.1, qRDW1.1, qRDW5.1, qSKC1.1, qRKC3, qRKC11.1, qSNC1.1, qRL2.1, qSNKR1.1, qCHLB3.1, qRNC3.1, qSES3.1	1, 2, 3, 5	Highest density at chromosome 1 with saltol locus	[74]
CSR10/PS5 140 F2	100 HvSSR	Total 39 QTLs for sodium content, potassium content, sodium/potassium ratio in roots and leaves, and grain yield	qNaL-1.2, qNa/KL-1.3, qKR-1 and qNa/KL-1.2 qGY-2, qGSSI-6.2	1, 2, 4, 6, 7, 8, 9, 10, 11, 12	Major QTLs identified for QTLs for sodium content, potassium content, sodium/potassium ratio, grain yield qGY-2, and SSI for grain yield qSSI-6.2	[75]
Wujiaozhan (WJZ)/Nipponbare 181 BC1F2	157 SSR	Germination rate and germination index	qGR6.1, qGR6.2, qGR8.1, qGR8.2, qGR10.1, qGR10.2, qGI6.2, qGI10.1, qGI10.2	6, 8, 10	Salt-tolerance-specific major QTL qGR6.2 was identified and fine-mapped.	[76]
CSR11/MI48 208	6,068 SNPs	New QTLs for grain yield under salt stress	qSSIGY2.1, qSSIGY2.2 and qSSIGY2.3	1, 2, 3, 5, 6, 9, 11 and 12	21 novel QTL for grain yield SSI	[77]
Weiguo/IR36 199 F2:3	KASP	25 KASP markers were used to narrow down the QTL region to 222 kb	qRSL7	7	A major QTL for relative shoot length (RSL) and candidate gene Os07g0569700 (OsSAP16) was indenitfied	[78]
MAGIC population 221 DC1	55k SNP array	Root length after salt stress (RLST), shoot length after salt stress (SLST), relative root length (RRL), dry shoot weight after salt stress (DSW), relative dry shoot weight (RDSW), biomass under salt stress (BST), relative biomass (RB)	qRLST5, qSLST1, qRRL2, qDSW9, qRDSW1, qBST9, qRB1	1, 2, 5, 9	7 QTLs delineated with 186 significant marker-trait associations were identified. A new QTL (qRRL2) at chromosome 2 for RRL and one multi-trait QTL for shoot length, root biomass, and root dry weight at chromosome 1 under salt stress	[79]
180 diverse genotypes	127 SSR	Twenty-eight marker-trait associations, among which 19 were identified for Na^+^, K^+^, Na^+^/K^+^ uptake in stem and leaves		1, 2, 3, 4, 5, 6, 7, 8, 9, 10, 11, 12		[80]
IR-44595 (indica)/IR- 318 (tropical japonica) 168 F2	2221 SNP	Na^+^ sheath-blade ratio, Na^+^ concentration in leaf blades, Na^+^ concentration in leaf sheaths, Na^+^ concentration in shoots, and K^+^/Na^+^ ratio in leaf blades, Na^+^ concentration in shoots and leaf sheath	qNSBR4, qNSBR11, qBNC11, qSHNC11, qSNC11, qBKNR11, qSNC4, qSNC1.1, qSNC1.2, At qSNC1.1, qSHNC1	1, 4, 11	two major QTLs (qNSBR4 and qNSBR11) were identified for Na^+^ sheath-blade ratio	[81]
Pokkali/I R29 80 RILs	206	High K^+^ absorption, low Na^+^ absorption and low Na^+^/K^+^ absorption ratio	Identified 10 QTLs: High K absorption: Chr1, 4 and 12; Na Absorption: chr1, 10, 3; Na-K ratio: Chr 1, 10, 12	1, 3, 4, 10, 12	Identified a major QTL Saltol on Chr1; Flanking AFLP marker P3/M9-8 and P1/M9-3	[82]
Pokkali/IR29 181 BC3F4	40 SSR	Salinity screening for percent survival and total leaf area affected at EC 18dS/m according to SES score	Identified 11 QTLs: Seven QTLs using single marker analysis (SMA) and six using the LTR-RSTEP, of which two were common	1, 3, 4, 5, 6, 10, 11	Similar salinity tolerance at the seedling stage without the Saltol allele distributed on Chr 5, 6, 10, 11 and three QTLs on Chr 3 with R2 value 8–15%	[83]
Milyang 23 (Indica) /Gihobyeo (Japonica) 164 F 18: F19 RILs	1300 RFLPs, SSLP, AFLP, isozyme	Seedling stage Salt tolerance in shoots at 0.5% and 0.7% NaCl concentration	Two QTLs: qST1, qST3	1, 3	qST1 and qST3 confer salt tolerance at young seedling stage explaining phenotypic variance 35–37%	[84]
Tarommhalli (Indica) /Khazar (Indica) 192 F2/F3	74 SSRs	Chlorophyll content, root and shoot length, fresh and dry weight of root and shoot, Na^+^ and K^+^ uptake, Na^+^/K^+^ ratio	Identified 32 QTLs; 11 major QTLs: qKUP-8, qKUP-3, qNAUP-1b, qDWR9a, qDWRO9b, qDWSH-3, qDWSH-7, qFWRO-3a, qFWSH-1, qFWSH-3, qSHL-3	1, 2, 3, 4, 5, 6, 7, 8, 9, 10	Two QTLs with largest effect, qDWRO-9a and qDWRO-9b for root dry weight explained 27.43 and 25.5% phenotypic variance	[85]
IR26/Jiucaiqing 150 F2:9 RILs		Imbibition rate and germination percentage at 100mM NaCl concentration	Identified 17 QTLs: qIR-4, qIR12, qIR-2, qIR-3, qIR-8, qIR10, qGP4-1, qGP4-2, qGP7-1, qGP-10, qIR-4, qIR-9, qIR-6, qGP7-2, qGP-2, qGP-3, qGP-9	2, 3, 4, 6, 7, 8, 9, 10, 12	QTLs for imbibition and germination were rarely co-located, and only one QTL qIR-3 and qGP-3 was located at the same position	[86]
Jiucaiqing (japonica)/IR26 150 F2:9 RILs	135 SSR	Na^+^ and K^+^ concentration in roots and shoots at 0, 100 and 120 mM NaCl concentration, salt tolerance rating	Identified 17 QTLs: qRKC6.1, qRKC6.2, qRKC10, qSKC10, qSNC9, qSKC1, qSKC9, qRKC4, qSNC11, qRKC10, qSTR7, qSNC3, qSKC1, qSKC4, qSKC9, qRKC4, qSNC11	1, 3, 4, 6, 7, 9, 10,11	One novel major QTL qSNC11 was identified explaining 16% phenotypic variance at the marker interval RM286-RM6288	[87]
Gharib (indica)/Sepidroud (indica) 148 F2:4	131 SSR 105 AFLP	Root and shoot: length, fresh weight, dry weight, biomass, shoot: Na^+^, K^+^ concentration and Na^+^/K^+^ ratio at the seedling stage, standard tolerance ranking (STR) from 0 to 9	Identified 41 QTLs on all rice chromosomes: Major effect QTLs: qRFW-4b, qSFW-4a, qSFW-5b, qSDW-2, qBM-5a, qBM-5b, qSTR-8, qSTR-9, qRL-9, qSHL-5, qSKC-1, qSKC-10b, qSNK-8, qCHL-8	All 12 chromosomes	Six QTLs were mapped for STR on chromosomes 1, 4, 8, 9, 11, and 12. Among these, two QTLs located on chromosome 8 (qSTR-8) and 9 (qSTR-9) had explained 19.66% and 21.7% of the total phenotypic variation.	[88]
Hasawi/BRR dhan 28 435 BC1F2	6209 SNP	Total 40 QTLs, including 24 plant height, productive tillers, panicle length, number of filled spikelets, number of unfilled spikelets, percent filled spikelets, grain yield, and Na^+^−K^+^ ratio.		1 to 12	3 important QTLs: qPT3.1 for productive tillers, qNFS3.1 for number of filled spikelets, qGY3.1 for grain yield	[89]
Horkuch/IR29 Biparental reciprocal population 137 F2:3	2230 SNP	Six QTLs for seedling stage shoot length, root length and total potassium	qSL.1, qSL.3, qSL.5, qRL.2, qTK.2, qTK.3, qPH.1, qPH.5, qET.7, qFGN.10, qFGW.10, qSF.10, qHI.10	1, 2, 3, 5, 7, 10	one large effect QTL for root length qRL.2, shoot length qSL.1 effective tiller number qET.7, filled grain weight qFGW.10, and spikelet fertility qSF.10	[90]
Akundi/BRRI dhan 49 F2:3	884 SNP	Seedling injury, Survival rate (%), shoot length, shoot dry weight, root length, Na^+^ and K^+^ concentration and Na^+^/K^+^ ratio.	q qSES1, qSES3, qSUR1, qSUR5.1, qSUR5.2, qSL1, qSDW5, qSDW11, qRL1, qSPAD12, qNa6, qK8, qK12, qNaKR8, qNaKR11	1, 3, 5, 6, 8, 11, 12	Three major QTLs: qSES3 for seedling injury, qNa6 sodium concentration, and qK8 for potassium concentration	[91]

**Table 2:** List of salt-tolerant rice varieties developed in different countries.

Country	Variety Name/Designation
Philippines	IRRI 112 as PSBRc48 (Hagonoy), IRRI 113 as PSBRc50 (Bicol), IRRI 124 as PSBRc84 (Sipocot), IRRI 125 as PSBRc86 (Matnog), IRRI 126 as PSBRc88 (Naga), IRRI 128 as NSICRc106, NSICRc296, NSICRc290, NSICRc294, NSIC2013Rc324, NSIC2013Rc326, NSIC2013Rc328, NSIC2013Rc330, NSIC2013Rc332, NSIC2013Rc334, NSIC2013Rc336, NSIC2013Rc338, NSIC2013Rc340
India	CSR10, CSR13, CSR22, CSR23, CSR27, CSR30 (Yamini), CSR36, Lunishree, Vytilla 1, Vytilla 2, Vytilla 3, Vytilla 4, Vyttila 5, Vyttila 6, Try 1, Panvel 1, Panvel 2, Panvel 3, Sumati, Jarava, Bhutnath, Usar dhan 1, Usar dhan 2, Usar dhan 3, CSR43,CR dhan405, CR dhan406
Bangladesh	BRRI dhan 40, BRRI dhan 41, BRRI dhan 55, BINA dhan10, BRRI dhan 61, BR11-*SalTol* *, BRRI dhan28-*SalTol* *, BRRI dhan 47 (Saltol) * (MAB product)
Vietnam	OM576, OM2717, OM2517, OM3242, AS996, OM5629, OM5981, OM6377, OM4488, OM11270, OM11271, Bacthom7-*SalTol* * (MAB product)
Egypt	Giza 177, Giza 178, Sakha 104, Sakha 111
Myanmar	Sangankhan Sinthwellat (Saltol) (MAS product)

* These ones were generated through the molecular breeding route.

## Data Availability

The data used in this work is available in the public domain.
